# Migrant patients living with HIV/AIDS in Japan: Review of factors associated with high dropout rate in a leading medical institution in Japan

**DOI:** 10.1371/journal.pone.0205184

**Published:** 2018-10-19

**Authors:** Mari Kinoshita, Shinichi Oka

**Affiliations:** AIDS Clinical Center, National Center for Global Health and Medicine, Shinjuku-ku, Tokyo, Japan; National and Kapodistrian University of Athens, GREECE

## Abstract

The present study aimed to identify factors associated with retention in HIV/AIDS care among migrant patients who visited the outpatient clinic of the AIDS Clinical Center, National Center for Global Health and Medicine in Tokyo, Japan. We reviewed the records of 551 selected (78 non-Japanese and 473 Japanese) patients who started visiting our clinic between 2011 and 2014. A total of 390 patients (70.8%: 38 non-Japanese and 352 Japanese) continued their visits during the study: from the date of their first visit to the end of 2015. The difference in retention rate was not significant (Incidence Rate Ratio (IRR) = 0.89, *p* = 0.27), but the loss-to-follow-up cases were considerably high among non-Japanese patients (n = 13, Incidence rate (IR) = 24.6 per 100,000 person-days, IRR = 3.65, p<0.01 after adjusting for time since diagnosis). The results showed, nevertheless, that there was no apparent association between retention and factors peculiar to non-Japanese. Twelve out of thirteen lost-to-follow-up non-Japanese patients held legal status to reside in Japan and were eligible for public health services. Nine had limited fluency in Japanese language, and six used alternative verbal communication. Further studies are needed to identify the factors responsible for the high dropout rate and to improve the care of migrant patients living with HIV/AIDS.

## Introduction

Migrants with HIV/AIDS account for 19.4% of 25,995 patients registered in Japan since 1985 [1]. Despite a marginal decrease in the number of newly reported cases in recent years, the number of new foreign cases remains stable, implying that the proportion of migrant patients is increasing [1]. Among newly reported migrant cases in 2016, 75% were non-AIDS HIV carriers [1], suggesting that early treatment and prevention of secondary infection are the focus of care at the hospital.

The initial impression of staff is a key determinant of retention in HIV care. Research shows that higher satisfaction with the initial visit correlates with better retention in care [2]. Furthermore, the initial communication at the hospital is considerably important among patients of foreign origin for successful establishment of HIV care. In this regard, the hospital is usually one of the most dominant sources of the necessary information. On the other hand, disappointment in service may force foreign patients to give up on treatment. Limited communication also makes the initial visit more difficult for patients of foreign origin.

The Japanese health policy guarantees provisions of essential medical care regardless of the patient’s country of origin as long as the beneficiaries cover the cost [3]. Although most of the main antiretroviral (ARV) drugs are readily available in Japan, the average cost of antiretroviral therapy (ART) is usually not affordable. A standard ART can cost as much as JPY 7,000 (equivalent to USD 70.00) per day [4]. Therefore, most of the patients cannot afford lifelong ART without the support of the Government-sponsored public medicare and the public health care cost subsidy for individuals with disabilities. The public health insurance (either National Health Insurance or Social Health Insurance for Laborers) covers 70% of the standard medical cost, and the subsidy for persons with disabilities, namely, “Jiritsu-Shien-Kosei-Iryou　身体障害者自立支援更生医療” or “Judo-Shin-Shin Shogaisya-Iryou-Hi-Josei 重度心身障害者医療費助成,” covers the rest. As long as the patient has legal status and a permanent visa in Japan, eligibility is defined solely by their physical status. If patients are eligible for the subsidy, they do not pay more than JPY 20,000 per month; the public support system pays the remainder [5].

A proportion of migrants is probably not aware of the above public aid systems because of the complexity of the application process at government offices, where the general staff does not speak foreign languages. Therefore, the hospital is usually the first gateway to accessing this information. Some non-Japanese patients may face particular difficulties, such as legal status or visa restrictions. Nevertheless, the top priority should be improving communication, because problems cannot be solved without an active communication gateway.

The AIDS Clinical Center (ACC), the National Center for Global Health and Medicine (NCGM), located in Tokyo, Japan, is one of the leading clinical research institutions of HIV/AIDS care and treatment. Since its establishment in 1997, over 4,014 patients with HIV/AIDS nationwide (15.4% of nationally registered cases) have visited the ACC to seek professional care and treatment. Migrant PLWHA formed approximately 10% of the patients in the past decade. The number of international visitors is expected to rise in line with global economic changes and the forthcoming Tokyo Olympic-Paralympic Games in 2020. Therefore, the ACC is preparing to improve their reception of foreign patients with HIV/AIDS. As part of these efforts, we use English or professional interpreters as alternative tools of communication. We proactively refer migrant patients to another facility when it is deemed appropriate, for example, for the availability of language service. We attempted to minimize the transfer time by arranging it as soon as we expected the completion of the initial phase assessment and treatment, expecting an early referral will reduce the risk of dropout.

The purpose of this study was to review the factors associated with retention of patients of foreign origin in HIV/AIDS care, and to evaluate the effectiveness of current approaches for migrant patients at our outpatient clinic (OPC).

## Methods

**Fig 1:**
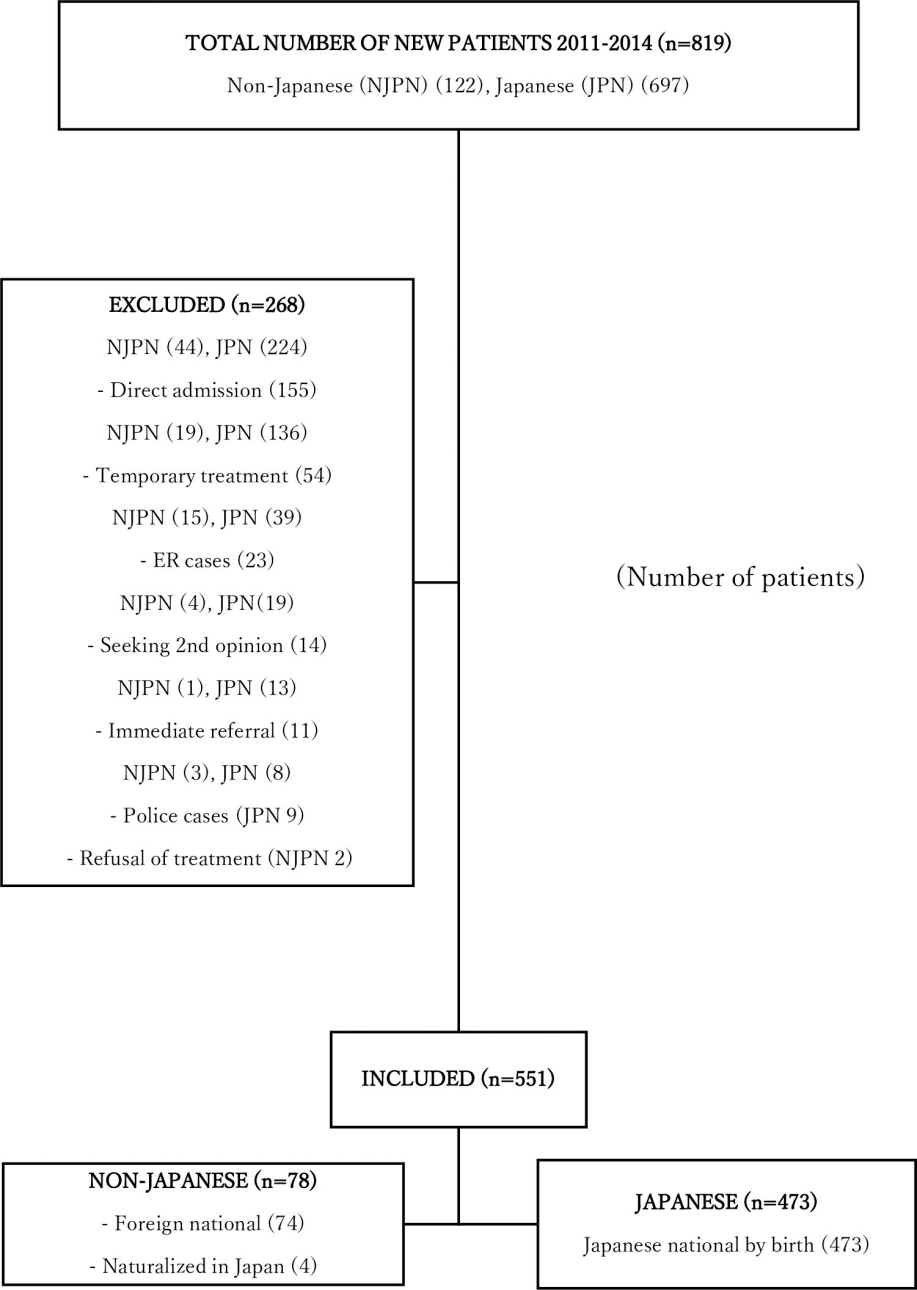
Flow diagram of patient selection.

Among the patients who visited our OPC for clinical consultation between January 2011 and December 2014, we chose those who sought long-term HIV/AIDS care and had passed through the conventional initial visit process. We excluded those patients who did not require the routinely-applied clinical care during the initial visit, for instance, emergency care, direct admission, same day medical referral to another health facility, second opinion, medical care for people in detention (police cases), or temporary treatment for other specialties (e.g., obstetrics/gynecology) (Fig 1). The same inclusion criteria applied to both non-Japanese and Japanese patients.

We define non-Japanese by the nationality at birth principle, regardless of their country of origin. Migrants naturalized in Japan are grouped in original nationality by birth. In such a half-Japanese case whereby dual citizenship is given on account of being born in Japan, we grouped them with the nationality acquired at the age of adulthood [6]. No originally Japanese national who was born and brought up abroad participated in the study. The group of non-Japanese in this study is identical to migrants who intend to reside in Japan permanently, as the study’s inclusion criteria outrightly excluded temporary visitors/travelers. We included migrants with expiring stay permits with a possibility of renewal or asylum seekers in the process of refugee status determination.

Based on the above criteria, we studied the records of 551 (78 migrant and 473 Japanese) patients. The records included both electronic versions and papers from the initial visit up to the end of 2015.

We defined retention as “the number of days from the initial date of visit to the last date of visit or December 31^st^, 2015 (the end of the study period) whichever came earlier”. We considered “lost-to-follow-up (LTF)” as those who do not show up for an appointment without prior notice for more than one year. We treated the cases that terminated our follow-up within and before the end of 2015 as “censored”. The reasons for censoring include transfer of care to another hospital or clinic, lost-to-follow-up, deaths and police custody.

The following data were collected from all participants: retention information (date of first visit, date of final visit, frequency of visits), data of patients’ physical conditions on the first visit (CD4+ lymphocyte cell count, HIV-1 ribonucleic acid (RNA) load, HIV stage, period of time between initial visit and the date of diagnosis, history of antiretroviral therapy (ART).

For all non-Japanese cases, information on country of origin, Japanese language proficiency, an alternative method of communication (use of English, interpreter), and public health insurance eligibility was gathered. Additional information was collected from non-Japanese LTF cases to review the factors surrounding their HIV disclosure to someone else in order to understand their support network and ART availability in their country of origin.

We used Microsoft Excel and STATA 10 (College Station, TX) for statistical analysis. The collected data on retention were processed as person-time data for incident rate (IR) and incident rate ratio (IRR) analysis using a log-rank test or simple and multiple Poisson regression analyses. Variables in multivariable models were selected based on the result of univariable analysis. Cox proportional hazard model was used to evaluate the level of risk reduction. We partly applied a two-sample test for binomial proportions to compare the difference of baseline characteristics between Non-Japanese and Japanese. We adopted a significance level of *p* less than 0.05 for statistical tests throughout the study.

The institutional ethics review board of the National Center for Global Health and Medicine (NCGM) approved the study (No.1452, 2015). Informed consent of participants was waived by the board because of the non-invasive design of the study.

## Results

Table 1 shows the baseline characteristics of the study population with comparison between non-Japanese and Japanese patients. The number of new non-Japanese and Japanese patients by year remained constant over the study period. The proportions of females, those diagnosed with HIV infection more than five years ago, those already on ART, and those with low HIV-1 RNA load were significantly high among non-Japanese patients (*p*<0.01, respectively). In other words, the proportions of males, recently diagnosed patients, treatment-naïve patients, and patients with over 1,000 copies/ml of viremia were high among Japanese patients.

**Table 1:** Baseline characteristics of study population, 2011–2014.

	Non-Japanese (n = 78)		Japanese (n = 473)		
Characteristics	n	%	n	%	*p*-value
Overall					
	78	14.2	473	85.8	-
Age					
0–19	1	1.3	5	1.1	0.877
20–29	23	26.5	109	23.0	0.499
30–39	30	38.5	188	39.8	0.828
40–49	19	24.4	124	26.2	0.737
50–59	5	6.4	32	6.8	0.896
60+	0	0.0	15	3.2	-
Gender					
Male	63	81.7	459	97.0	<0.001
Female	15	18.3	14^a^	3.0	<0.001
Year of first visit					
2011	22	30.5	122	25.8	0.383
2012	15	18.3	131	27.7	0.081
2013	21	25.6	116	24.5	0.835
2014	20	25.6	104	22.0	0.481
Time since HIV diagnosis					
< 1 year	53	68.0	394	83.3	<0.001
1 to 5 year	14	18.0	52	11.0	0.779
> 5 year	11	14.0	27	5.7	0.007
On ART					
Yes	20	25.6	53	11.2	<0.001
No	58	74.4	420	88.8	<0.001
CD4+ cell count					
< 200 /μl	19	25.6	152	32.1	0.251
≥ 200 /μl	59	74.4	321	67.9	0.251
HIV-1 RNA					
< 1000 copies/ml	21	25.6	52	11.0	<0.001
≥ 1000 copies/ml	57	74.4	421	89.0	<0.001
HIV stage					
AIDS	11	14.1	80	16.9	0.536
AC	47	60.3	356	75.3	0.006
Symptomatic	8	10.3	23	4.9	0.055
Others	12	15.4	14	3.0	<0.001

All data are as of first visit.

Female data include a Male-To-Female transgender.

AC stands for Asymptomatic Careers.

Table 2 picked up some migrant-specific factors to describe characteristics of non-Japanese patients’ group. They were mostly from Asia (n = 37, 47.4%), reflecting geographical proximity, followed by Africa (n = 16, 20.5%) and North America (n = 9, 11.5%). There was no significant change in the proportion of patients according to regions of origin over the study period, except a marginal decrease in the number of new cases from the African region. China (including Taipei China) was the top country/territory of origin over the study period (n = 13, 16.7%). The number of new patients from China has remarkably increased since 2014, which is possibly in line with the global economic conditions and relaxation of Japanese immigration policy for an entry visa to Chinese citizens [7].

**Table 2:** Profiles of non-Japanese participants (n = 78).

	n	%
Region of origin		
Asia	37	47.4
Africa	16	20.5
North America	9	11.5
Latin America	7	9.0
Europe	6	7.7
Oceania	3	3.9
National Health Insurance		
Eligible	71	91.0
Not Eligible	7	9.0
Japanese Language Proficiency		
Fluent/Native	21	26.9
Conversation	27	34.6
Poor/Words	29	37.2
Unknown	1	1.3
Alternative Communication with Hospital Staff		
Use of English	47	59.5
Use of Interpreter	6	7.6

Table 2 also shows Japanese language proficiency by migrant patients. We categorized the level of speaking/listening ability into six levels: poor, words-only, conversational, fluent, native, and unknown. We considered the level [poor] to level [conversational] as insufficient for essential communication at the hospital, as they may not be adequate for patients to understand their usually complex medical condition. Based on these criteria, 71.8% (n = 56) had experienced incomplete communication on their own. English appeared to be the most frequently used language for basic interpersonal communication skills (BICS) during the regular visits to the hospital. Twenty-four out of the 78 migrant patients were from a country/territory where English was one of the native languages [8].

Regarding alternative modes of communication used by patients of foreign origin, 47 (60.0%) attempted direct conversation with staff using English, while six (7.6%) were accompanied by professional interpreters. During the study period, six used a charge-free professional interpreting service provided by the local government or charity/non-profit organizations in Spanish, Portuguese, Chinese, and Thai languages. There was no record of pay service use. Very few patients used professional interpreters on their initial visit. In most cases interpreters/counselors were arranged by the hospital staff and used for subsequent visits.

Table 3 shows the outcomes of the follow-up by incidence rate stratified by groups and factors. A total of 390 patients maintained their visits to the hospital over the study period, while 161 patients were censored. Further analysis suggested that the difference in retention rate was not significantly different between non-Japanese and Japanese (IR[non-Japanese] = 71.9 per 100,000 person-days, IR[Japanese] = 80.1 per 100,000 person-days, IRR = 0.89, *p* = 0.27).

**Table 3:** Follow-up outcome between non-Japanese and Japanese patients.

	Non-Japanese (n = 78)						Japanese (n = 473)						IRR of LTF
Characteristics		Retained	Referral	Police	Death	LTF		Retained	Referred	Police	Death	LTF	
	person-days	Count (IR per 100,000 person-days)					person-days	Count (IR per 100,000 person-days)					
Overall													
	52,872	38 (71.9)	27 (51.1)	0 (0.0)	0 (0.0)	13 (24.6)	439,456	352 (80.1)	97 (22.1)	3 (0.0)	3 (0.0)	18 (4.1)	6.0**
Age, years old													
0–29	13,002	6 (46.2)	14 (107.7)	-	-	4 (30.8)	89,161	70 (78.5)	36 (40.4)	0 (0.0)	0 (0.0)	8 (9.0)	3.43*
30–39	15,641	12 (76.7)	12 (76.7)	-	-	6 (38.4)	174,887	135 (77.2)	45 (25.7)	2 (1.1)	0 (0.0)	6 (3.4)	11.2**
40–49	19,924	16 (80.3)	1 (5.0)	-	-	2 (10.0)	123,830	108 (87.2)	11 (8.9)	1 (0.0)	1 (0.0)	3 (2.4)	4.1
50+	4,305	4 (92.9)	0 (0.0)	-	-	1 (23.2)	51,578	39 (75.6)	5 (9.7)	0 (0.0)	2 (3.9)	1 (1.9)	12.0
Gender													
Male	39,002	30 (76.9)	22 (56.4)	-	-	11 (28.2)	423,793	339 (80.0)	97 (22.9)	3 (0.0)	3 (0.0)	17 (4.0)	7.0**
Female	13,870	8 (57.7)	5 (36.1)	-	-	2 (14.4)	15,663	13 (83.0)	0 (0.0)	0 (0.0)	0 (0.0)	1 (6.4)	2.3
Year of first visit													
2011	24,376	11 (45.1)	7 (28.7)	-	-	4 (16.4)	168,456	90 (53.4)	25 (14.8)	1 (0.0)	2 (1.2)	4 (2.4)	6.9**
2012	11,544	7 (60.6)	6 (52.0)	-	-	2 (17.3)	133,422	92 (69.0)	32 (24.0)	0 (0.0)	1 (0.0)	6 (4.5)	3.9
2013	8,666	7 (80.8)	8 (92.3)	-	-	6 (69.2)	87,724	86 (98.0)	25 (28.5)	1 (1.1)	0 (0.0)	4 (4.6)	15.2**
2014	8,286	13 (156.9)	6 (72.4)	-	-	1 (12.1)	49,854	84 (168.5)	15 (30.1)	1 (2.0)	0 (0.0)	4 (8.0)	1.5
Time since diagnosis													
0–1 year	37,162	29 (78.0)	19 (51.1)	-	-	5 (13.5)	371,945	302 (81.2)	74 (19.9)	1 (0.0)	2 (0.0)	15 (4.3)	3.3*
1–5 years	9,395	5 (53.2)	7 (74.5)	-	-	2 (21.3)	45,233	32 (70.7)	16 (35.4)	2 (4.4)	1 (2.2)	1 (2.2)	9.6*
5 years+	6,315	4 (63.3)	1 (15.8)	-	-	6 (95.0)	22,278	18 (80.8)	7 (31.4)	0 (0.0)	0 (0.0)	2 (9.0)	10.6**
On ART													
Yes	9,549	5 (76.2)	9 (94.3)	-	-	6 (62.8)	45,079	36 (79.9)	12 (26.6)	2 (4.4)	0 (0.0)	3 (6.7)	9.4**
No	43,323	33 (52.4)	18 (41.6)	-	-	7 (16.2)	394,377	316 (80.1)	85 (21.6)	1 (0.0)	3 (0.0)	15 (3.8)	4.3**
CD4+ cell, count//μl													
< 200	15,018	15 (99.9)	2 (13.3)	-	-	2 (13.3)	146,988	123 (83.7)	23 (15.7)	0 (0.0)	0 (0.0)	6 (4.1)	3.3
≥ 200	37,854	23 (60.8)	25 (66.0)	-	-	11 (29.1)	292,468	229 (78.3)	74 (25.3)	3 (1.0)	3 (1.0)	12 (3.2)	7.1**
HIV-1 RNA, copies/ml													
< 1000	11,670	7 (60.0)	9 (77.1)	-	-	5 (42.8)	46,207	35 (75.8)	12 (26.0)	2 (4.3)	0 (0.0)	3 (6.5)	6.6**
≥ 1000	41,202	31 (75.2)	18 (43.7)	-	-	8 (19.4)	393,249	317 (80.6)	85 (21.6)	1 (0.0)	3 (0.0)	15 (3.8)	5.1**
HIV stage													
AIDS	7,980	8 (100.3)	2 (25.1)	-	-	1 (12.5)	82,204	260 (81.5)	78 (9.7)	2 (1.2)	2 (1.2)	3 (3.7)	3.4
AC	29,920	20 (66.8)	19 (63.5	-	-	8 (26.7)	337,746	67 (77.0)	8 (23.1)	1 (0.0)	1 (0.0)	14 (4.2)	6.5**
Symptomatic	9,316	6 (64.4)	0 (0.0)	-	-	2 (21.5)	14,855	20 (134.6)	3 (20.2)	0 (0.0)	0 (0.0)	0 (0.0)	NA
Others	5,656	4 (70.7)	6 (106.1)	-	-	2 (35.4)	4,651	5 (107.5)	8 (172.0)	0 (0.0)	0 (0.0)	1 (2.2)	1.6

Characteristics as of the first visit. Japanese Female data include a Male-To-Female transgender.

IR stands for Incidence Rate per 100,000 person-days. IRR stands for Incident Rate Ratio. AC stands for Asymptomatic Carriers.

* p<0.05

** p<0.01.

The primary reason for censoring is referral to another clinical facility. All 97 referrals of Japanese were addressed to hospitals and clinics in Japan, while eight out of 27 non-Japanese cases were addressed to an unspecified recipient in their home countries, as they returned to their country of origin.

The second major reason for censoring was LTF (31 cases). The overall LTF rate was significantly higher among migrant patients compared to Japanese Patients (IRR = 6.0, *p*<0.01)

To analyze the factors associated with high LTF among non-Japanese patients, we conducted regression analysis using Poisson regression model. Table 4 shows the results of analysis of the associations among sociocultural factors and LTF in migrant patients. Univariate regression results suggested that the incidence rate of LTF increased 4.38 times among non-Japanese (*p*<0.01), 2.68 times among patients on ART in the initial visit (*p* = 0.01), and 1.37 times with each additional year since the time of diagnosis (*p*<0.01). On the other hand, LTF rate decreased by 56% among those with over 1,000 copies/ml of HIV-1 RNA (*p* = 0.05).

**Table 4:** Results of Poisson regression analyses (n = 551).

	Univariable Analysis		Multivariable Model (A)		Multivariable Model (B)	
Variables	IRR (95%CI)	*p*-value	*IRR (95% CI)*	*p-value*	*IRR (95% CI)*	*p-value*
Non-Japanese	4.38 (2.15–8.94)	<0.01	3.60 (1.72–7.51)	<0.01	3.65 (1.76–7.59)	<0.01
Age at enrolment	0.97 (0.93–1.00)	0.11				
Male gender	0.52 (0.16–1.70)	0.28				
Year of enrolment	0.96 (0.70–1.32)	0.80				
On ART	2.68 (1.23–5.82)	0.01	1.16 (0.37–3.68)	0.79		
CD4+ cell count≥200/μl	1.29 (0.58–2.89)	0.53				
HIV-1 RNA>1000 copies/ml	0.44 (0.20–0.98)	0.05	0.96 (0.33–2.81)	0.95		
Time since HIV diagnosis, year	1.37 (1.16–1.61)	<0.01	1.26 (1.03–1.55)	0.03	1.29 (1.09–1.53)	<0.01

All variables with a *p*<0.1 in the univariable analyses were submitted in multivariable model (A). Likewise, all variables with a *p*<0.1 in model (A) were submitted in the model (B)

There was no significant difference detected in gender, year of first visit (enrollment into the study), CD4+ cell count at initial visit.

By submitting variables with *p*<0.1 in the univariable analysis and multivariable analysis, we came up with a regression model with variables of a difference of non-Japanese/Japanese and time since diagnosis. The estimated LTF rate was increased 3.65 times among non-Japanese patients (IRR = 3.65, 95%CI: 1.76–7.59, *p*<0.01, Table 4.).

In addition, we have conducted an analysis using Cox Hazard Model to evaluate the effect of alternative communication tools (e.g. the use of English and an interpreter). We detected little evidence of risk reduction in the use of English and the use of an interpreter for communication with hospital staff (hazard ratio = 0.58, *p* = 0.32, hazard ratio = 1.87, *p* = 0.42, respectively).

Among 31 cases who were lost-to-follow-up within the study period, Table 5 picked up the profiles of the 13 non-Japanese cases. Many of these cases had limited proficiency in Japanese language. Six out of the 13 used English as an alternative mode of communication, and only a few used professional interpreters. All but one had legal status in Japan with a valid stay permit at the time of the initial visit, which implies that 12 (with one exception) were deemed eligible for public health insurance. It was anticipated that disclosure of their HIV status to friends or partners would help with continuity of their follow-up. A few had disclosed their status, but were still lost-to-follow-up. Some patients were from countries that offered free ART, so it was considered possible that they could have received treatment if they had gone back to their country. We hoped that they had returned to their countries of origin; however, their whereabouts are unknown.

**Table 5:** Profiles of the non-Japanese patients who were lost-to-follow-up during the study period (n = 13).

Case	Time to attrition (Days)	Gender	CD4+ count/μl at first visit	Japanese language proficiency	English use	Interpreter use	Disclosure of HIV	Legal status	Region of origin	ART in home country^1)^^–^^3)^
1	1	M	344	Conversation	Yes	No	Yes	Yes	Asia	Subsidized
2	7	M	341	Conversation	Yes	No	None	Yes	Oceania	Free
3	29	M	282	Conversation	Yes	Yes	Yes	Yes	Latin America	Free
4	29	M	637	Fluent	No	No	Unknown	Yes	Latin America	Free
5	32	F	423	Words only	Yes	No	None	Yes	Africa	Free
6	43	M	181	Words only	No	No	None	No	Africa	No
7	85	M	275	Poor	No	No	Yes	Yes	Africa	No
8	107	M	373	Fluent	No	No	None	Yes	Asia	Free
9	135	M	322	Poor	Yes	No	None	Yes	North America	Conditional
10	482	M	291	Native	No	No	Yes	Yes	Asia	Subsidized
11	644	F	773	Conversation	No	No	Yes	Yes	Asia	Subsidized
12	680	M	280	Conversation	Yes	No	No	Yes	Europe	Free
13	1180	M	135	Fluent	No	No	Yes	Yes	Asia	Subsidized

^1)^WHO; Countries offering free access to HIV treatment; Developing Countries & Free Access Fact Sheet; December 2005.

^2)^WHO; Democratic Republic of the Congo; Summary Country Profile for HIV/AIDS Treatment Scale-Up; December 2005.

^3)^UNAIDS; Access to antiretroviral therapy in Africa; status report on progress towards the 2015 targets; 2013.

## Discussion

Among the study population, we did not detect a difference in retention rate between migrant patients and Japanese patients. On the other hand, we have detected a significant increase in censoring among non-Japanese. The discordant result may be explained by the timing of censoring. The primary reasons for censoring were the transfer of care to another hospital/clinic and LTF. As we mentioned earlier, we try to transfer migrant patients to another hospital/clinic as soon as we finish the initial medical assessment. We also have results that many of the loss-to-follow-up cases occurred within 90 days following the first visit. Therefore, if the event of censoring occurred relatively earlier among migrant patients, the incident rate would be increased significantly.

An early dropout generates concern that some patients stopped visiting our center before we completed their initial assessment and ensured the provision of essential information. It highlights the difficulty in the establishment of care compared to its continuation [9]. Providing clinical care to people living with HIV/AIDS (PLWHA) is important from a public health point of view. Failure to establish HIV/AIDS care and leaving patients untreated is a potential risk of secondary infection with the possible spread of the virus to families or communities unless the patients return to their home country to seek treatment.

Adequate provision of social support information in the first stage of the visit is therefore critical for retention of patients of foreign origin in continuous HIV/AIDS care [10]. This information must emphasize the availability of public support systems to all patients, regardless of their country of origin. Those who are from a country where the government ill-treats PLWHA may be overly concerned about how the Japanese government will treat them as aliens. Case-specific information may also be necessary, such as support options available for asylum seekers (unrecognized refugees), long-term care options and age-related welfare services available in Japan for permanent-stay permit holders.

The high loss-to-follow-up rate among migrant patients is in line with previous studies that suggested an increased risk of non-adherence to ART among immigrant patients [11–14]. Some of the extant literature in this field indicated that limited social support is the primary factor for the dropout. If patients are not eligible for the government subsidy and their levels of social support become weak, they are then unlikely to adhere to the treatment [15]. However, there are conflicting results in this study that suggest the loss-to-follow-up rate was not statistically significant for legal status. Moreover, most of the loss-to-follow-up cases were eligible for government assistance. Therefore, we cannot determine at this stage whether legal status was the main reason for the high dropout rate among migrant patients. The cause of loss-to-follow-up is not likely to be an actual rejection of social service applications, but rather the fear of rejection because of social status as a foreigner. If individuals are unaware of their eligibility for social service, they may give up on treatment out of fear that they cannot afford it.

Another possible cause of loss-to-follow-up among migrant patients is poor patient-staff communication and subsequent lack of social support information. Although the results of this research did not provide substantial evidence, many of the early loss-to-follow-up cases were not fluent in the Japanese language. No doubt it is hard to build patient–staff trust and to determine whether Japanese language proficiency is insufficient, which is crucial to establishing HIV/AIDS care [16]. Thus, limited verbal communication between hospital staff and patients appeared to be one of the factors responsible for the low retention rate.

Some of the patients used alternative methods of communication, such as using English for direct communication, or the help of a professional interpreter. Despite the effort of alternative communication, we did not detect a significant risk reduction in drop-out through the alternative modes of communication. The small sample size may not be the only reason for this. First, English is usually the second language for both migrant patients and hospital staff. Second, the availability of translation tools is limited to some charge-free services because ACC does not have a specific budget allocation to cover the cost of pay service.

We sometimes experience a service preference gap. Hospital staff prefer a face-to-face interpretation service as they believe it to be more reliable, convenient, and flexible, but some patients prefer a telephone interpreter service to enable them to hide their identity from the translator who is sometimes an acquaintance of the patient. Some patients refuse to seek the help of native speakers as they fear it will jeopardize their confidentiality among resident colleagues in Japan, particularly when their local community is small. The challenge is to find the means to ensure reliability and confidentiality of the translation service to both patients and hospital staff. The use of professional interpretation is more promising compared to the unreliable use of English, because the hospital-wide telephone interpretation service has recently become available in several languages, and the choice is available to more people.

Although previous studies identified several factors as possibly being associated with retention in care [17–21], we did not collect the following information: local support networks for migrant patients, mental health conditions, socioeconomic status, transportation, housing, and food security. We detected an association between early LTF and the time period since HIV diagnosis, there was little evidence of an association with retention. Further research is needed in this field since our study did not detect other factors associated with loss-to-follow-up among migrant patients.

## Conclusion

The results of the study suggested a significantly higher dropout rate of migrant patients with HIV/AIDS who sought care at the hospital compared to that of Japanese patients. Nevertheless, we did not detect an apparent association between retention and the factors peculiar to migrants. There was no evidence of risk reduction owing to the current communication assistance (e.g. interpretation service) that we are providing. Further studies are needed to analyze factors of high dropout and address the issue of migrant PLWHA to improve retention in HIV/AIDS care.

## Supporting information

S1 Dataset (PDF)
