# Supplementary material for: Migrant patients living with HIV/AIDS in Japan: Review of factors associated with high dropout rate in a leading medical institution in Japan
Source: PLoS One. 2018 Oct 19;13(10):e0205184. doi: 10.1371/journal.pone.0205184 (PMC6195273; doi:10.1371/journal.pone.0205184)
Supplement: S1 Dataset — (PDF) [file pone.0205184.s001.pdf]

**S1 Dataset.**

| Sr | Origin | Follow-up days | Outcome |
|---|---|---|---|
| 1 | Non-Japanese | 1180 | Lost-to-follow-up |
| 2 | Non-Japanese | 680 | Lost-to-follow-up |
| 3 | Non-Japanese | 107 | Lost-to-follow-up |
| 4 | Non-Japanese | 32 | Lost-to-follow-up |
| 5 | Non-Japanese | 29 | Lost-to-follow-up |
| 6 | Non-Japanese | 1 | Lost-to-follow-up |
| 7 | Non-Japanese | 482 | Lost-to-follow-up |
| 8 | Non-Japanese | 135 | Lost-to-follow-up |
| 9 | Non-Japanese | 43 | Lost-to-follow-up |
| 10 | Non-Japanese | 644 | Lost-to-follow-up |
| 11 | Non-Japanese | 7 | Lost-to-follow-up |
| 12 | Non-Japanese | 29 | Lost-to-follow-up |
| 13 | Non-Japanese | 85 | Lost-to-follow-up |
| 14 | Non-Japanese | 1786 | OK |
| 15 | Non-Japanese | 1744 | OK |
| 16 | Non-Japanese | 1744 | OK |
| 17 | Non-Japanese | 1736 | OK |
| 18 | Non-Japanese | 1712 | OK |
| 19 | Non-Japanese | 1710 | OK |
| 20 | Non-Japanese | 1619 | OK |
| 21 | Non-Japanese | 1571 | OK |
| 22 | Non-Japanese | 1551 | OK |
| 23 | Non-Japanese | 1507 | OK |
| 24 | Non-Japanese | 1501 | OK |
| 25 | Non-Japanese | 1444 | OK |
| 26 | Non-Japanese | 1442 | OK |
| 27 | Non-Japanese | 1435 | OK |
| 28 | Non-Japanese | 1376 | OK |
| 29 | Non-Japanese | 1212 | OK |
| 30 | Non-Japanese | 1185 | OK |
| 31 | Non-Japanese | 1142 | OK |
| 32 | Non-Japanese | 1029 | OK |
| 33 | Non-Japanese | 1024 | OK |
| 34 | Non-Japanese | 991 | OK |
| 35 | Non-Japanese | 849 | OK |
| 36 | Non-Japanese | 843 | OK |
| 37 | Non-Japanese | 841 | OK |
| 38 | Non-Japanese | 833 | OK |

**S1 Dataset.**

| Sr | Origin | Follow-up days | Outcome |
|---|---|---|---|
| 39 | Non-Japanese | 681 | OK |
| 40 | Non-Japanese | 661 | OK |
| 41 | Non-Japanese | 645 | OK |
| 42 | Non-Japanese | 637 | OK |
| 43 | Non-Japanese | 567 | OK |
| 44 | Non-Japanese | 547 | OK |
| 45 | Non-Japanese | 522 | OK |
| 46 | Non-Japanese | 508 | OK |
| 47 | Non-Japanese | 456 | OK |
| 48 | Non-Japanese | 435 | OK |
| 49 | Non-Japanese | 430 | OK |
| 50 | Non-Japanese | 416 | OK |
| 51 | Non-Japanese | 375 | OK |
| 52 | Non-Japanese | 281 | Referred |
| 53 | Non-Japanese | 430 | Referred |
| 54 | Non-Japanese | 190 | Referred |
| 55 | Non-Japanese | 295 | Referred |
| 56 | Non-Japanese | 42 | Referred |
| 57 | Non-Japanese | 1081 | Referred |
| 58 | Non-Japanese | 542 | Referred |
| 59 | Non-Japanese | 94 | Referred |
| 60 | Non-Japanese | 113 | Referred |
| 61 | Non-Japanese | 73 | Referred |
| 62 | Non-Japanese | 241 | Referred |
| 63 | Non-Japanese | 11 | Referred |
| 64 | Non-Japanese | 14 | Referred |
| 65 | Non-Japanese | 40 | Referred |
| 66 | Non-Japanese | 372 | Referred |
| 67 | Non-Japanese | 116 | Referred |
| 68 | Non-Japanese | 525 | Referred |
| 69 | Non-Japanese | 256 | Referred |
| 70 | Non-Japanese | 45 | Referred |
| 71 | Non-Japanese | 45 | Referred |
| 72 | Non-Japanese | 154 | Referred |
| 73 | Non-Japanese | 622 | Repatriation |
| 74 | Non-Japanese | 475 | Repatriation |
| 75 | Non-Japanese | 1850 | Repatriation |
| 76 | Non-Japanese | 375 | Repatriation |

**S1 Dataset.**

| Sr | Origin | Follow-up days | Outcome |
|---|---|---|---|
| 77 | Non-Japanese | 77 | Repatriation |
| 78 | Non-Japanese | 352 | Repatriation |
| 79 | Japanese | 1768 | Death |
| 80 | Japanese | 1135 | Death |
| 81 | Japanese | 387 | Death |
| 82 | Japanese | 1368 | Lost-to-follow-up |
| 83 | Japanese | 751 | Lost-to-follow-up |
| 84 | Japanese | 3 | Lost-to-follow-up |
| 85 | Japanese | 483 | Lost-to-follow-up |
| 86 | Japanese | 1188 | Lost-to-follow-up |
| 87 | Japanese | 26 | Lost-to-follow-up |
| 88 | Japanese | 62 | Lost-to-follow-up |
| 89 | Japanese | 1192 | Lost-to-follow-up |
| 90 | Japanese | 19 | Lost-to-follow-up |
| 91 | Japanese | 648 | Lost-to-follow-up |
| 92 | Japanese | 28 | Lost-to-follow-up |
| 93 | Japanese | 644 | Lost-to-follow-up |
| 94 | Japanese | 81 | Lost-to-follow-up |
| 95 | Japanese | 115 | Lost-to-follow-up |
| 96 | Japanese | 14 | Lost-to-follow-up |
| 97 | Japanese | 29 | Lost-to-follow-up |
| 98 | Japanese | 99 | Lost-to-follow-up |
| 99 | Japanese | 120 | Lost-to-follow-up |
| 100 | Japanese | 1820 | OK |
| 101 | Japanese | 1814 | OK |
| 102 | Japanese | 1810 | OK |
| 103 | Japanese | 1809 | OK |
| 104 | Japanese | 1803 | OK |
| 105 | Japanese | 1799 | OK |
| 106 | Japanese | 1795 | OK |
| 107 | Japanese | 1795 | OK |
| 108 | Japanese | 1795 | OK |
| 109 | Japanese | 1792 | OK |
| 110 | Japanese | 1788 | OK |
| 111 | Japanese | 1782 | OK |
| 112 | Japanese | 1780 | OK |
| 113 | Japanese | 1771 | OK |
| 114 | Japanese | 1768 | OK |

**S1 Dataset.**

| Sr | Origin | Follow-up days | Outcome |
|---|---|---|---|
| 115 | Japanese | 1764 | OK |
| 116 | Japanese | 1764 | OK |
| 117 | Japanese | 1760 | OK |
| 118 | Japanese | 1760 | OK |
| 119 | Japanese | 1754 | OK |
| 120 | Japanese | 1753 | OK |
| 121 | Japanese | 1733 | OK |
| 122 | Japanese | 1717 | OK |
| 123 | Japanese | 1716 | OK |
| 124 | Japanese | 1712 | OK |
| 125 | Japanese | 1711 | OK |
| 126 | Japanese | 1709 | OK |
| 127 | Japanese | 1698 | OK |
| 128 | Japanese | 1694 | OK |
| 129 | Japanese | 1689 | OK |
| 130 | Japanese | 1683 | OK |
| 131 | Japanese | 1677 | OK |
| 132 | Japanese | 1670 | OK |
| 133 | Japanese | 1667 | OK |
| 134 | Japanese | 1663 | OK |
| 135 | Japanese | 1656 | OK |
| 136 | Japanese | 1653 | OK |
| 137 | Japanese | 1647 | OK |
| 138 | Japanese | 1647 | OK |
| 139 | Japanese | 1641 | OK |
| 140 | Japanese | 1640 | OK |
| 141 | Japanese | 1633 | OK |
| 142 | Japanese | 1633 | OK |
| 143 | Japanese | 1632 | OK |
| 144 | Japanese | 1631 | OK |
| 145 | Japanese | 1610 | OK |
| 146 | Japanese | 1606 | OK |
| 147 | Japanese | 1606 | OK |
| 148 | Japanese | 1592 | OK |
| 149 | Japanese | 1592 | OK |
| 150 | Japanese | 1578 | OK |
| 151 | Japanese | 1577 | OK |
| 152 | Japanese | 1572 | OK |

**S1 Dataset.**

| Sr | Origin | Follow-up days | Outcome |
|---|---|---|---|
| 153 | Japanese | 1571 | OK |
| 154 | Japanese | 1563 | OK |
| 155 | Japanese | 1562 | OK |
| 156 | Japanese | 1556 | OK |
| 157 | Japanese | 1556 | OK |
| 158 | Japanese | 1555 | OK |
| 159 | Japanese | 1551 | OK |
| 160 | Japanese | 1550 | OK |
| 161 | Japanese | 1542 | OK |
| 162 | Japanese | 1541 | OK |
| 163 | Japanese | 1537 | OK |
| 164 | Japanese | 1535 | OK |
| 165 | Japanese | 1534 | OK |
| 166 | Japanese | 1528 | OK |
| 167 | Japanese | 1526 | OK |
| 168 | Japanese | 1526 | OK |
| 169 | Japanese | 1522 | OK |
| 170 | Japanese | 1515 | OK |
| 171 | Japanese | 1509 | OK |
| 172 | Japanese | 1508 | OK |
| 173 | Japanese | 1508 | OK |
| 174 | Japanese | 1507 | OK |
| 175 | Japanese | 1507 | OK |
| 176 | Japanese | 1507 | OK |
| 177 | Japanese | 1502 | OK |
| 178 | Japanese | 1499 | OK |
| 179 | Japanese | 1498 | OK |
| 180 | Japanese | 1498 | OK |
| 181 | Japanese | 1494 | OK |
| 182 | Japanese | 1481 | OK |
| 183 | Japanese | 1478 | OK |
| 184 | Japanese | 1478 | OK |
| 185 | Japanese | 1477 | OK |
| 186 | Japanese | 1474 | OK |
| 187 | Japanese | 1474 | OK |
| 188 | Japanese | 1471 | OK |
| 189 | Japanese | 1467 | OK |
| 190 | Japanese | 1456 | OK |

**S1 Dataset.**

| Sr | Origin | Follow-up days | Outcome |
|---|---|---|---|
| 191 | Japanese | 1451 | OK |
| 192 | Japanese | 1451 | OK |
| 193 | Japanese | 1444 | OK |
| 194 | Japanese | 1443 | OK |
| 195 | Japanese | 1443 | OK |
| 196 | Japanese | 1439 | OK |
| 197 | Japanese | 1435 | OK |
| 198 | Japanese | 1431 | OK |
| 199 | Japanese | 1430 | OK |
| 200 | Japanese | 1425 | OK |
| 201 | Japanese | 1424 | OK |
| 202 | Japanese | 1422 | OK |
| 203 | Japanese | 1421 | OK |
| 204 | Japanese | 1417 | OK |
| 205 | Japanese | 1417 | OK |
| 206 | Japanese | 1416 | OK |
| 207 | Japanese | 1416 | OK |
| 208 | Japanese | 1407 | OK |
| 209 | Japanese | 1400 | OK |
| 210 | Japanese | 1400 | OK |
| 211 | Japanese | 1389 | OK |
| 212 | Japanese | 1387 | OK |
| 213 | Japanese | 1386 | OK |
| 214 | Japanese | 1376 | OK |
| 215 | Japanese | 1372 | OK |
| 216 | Japanese | 1369 | OK |
| 217 | Japanese | 1365 | OK |
| 218 | Japanese | 1361 | OK |
| 219 | Japanese | 1360 | OK |
| 220 | Japanese | 1360 | OK |
| 221 | Japanese | 1355 | OK |
| 222 | Japanese | 1354 | OK |
| 223 | Japanese | 1351 | OK |
| 224 | Japanese | 1347 | OK |
| 225 | Japanese | 1346 | OK |
| 226 | Japanese | 1326 | OK |
| 227 | Japanese | 1325 | OK |
| 228 | Japanese | 1319 | OK |

**S1 Dataset.**

| Sr | Origin | Follow-up days | Outcome |
|---|---|---|---|
| 229 | Japanese | 1316 | OK |
| 230 | Japanese | 1313 | OK |
| 231 | Japanese | 1311 | OK |
| 232 | Japanese | 1303 | OK |
| 233 | Japanese | 1302 | OK |
| 234 | Japanese | 1292 | OK |
| 235 | Japanese | 1290 | OK |
| 236 | Japanese | 1288 | OK |
| 237 | Japanese | 1284 | OK |
| 238 | Japanese | 1282 | OK |
| 239 | Japanese | 1267 | OK |
| 240 | Japanese | 1253 | OK |
| 241 | Japanese | 1246 | OK |
| 242 | Japanese | 1243 | OK |
| 243 | Japanese | 1240 | OK |
| 244 | Japanese | 1239 | OK |
| 245 | Japanese | 1235 | OK |
| 246 | Japanese | 1235 | OK |
| 247 | Japanese | 1226 | OK |
| 248 | Japanese | 1226 | OK |
| 249 | Japanese | 1226 | OK |
| 250 | Japanese | 1220 | OK |
| 251 | Japanese | 1220 | OK |
| 252 | Japanese | 1213 | OK |
| 253 | Japanese | 1207 | OK |
| 254 | Japanese | 1199 | OK |
| 255 | Japanese | 1192 | OK |
| 256 | Japanese | 1191 | OK |
| 257 | Japanese | 1191 | OK |
| 258 | Japanese | 1187 | OK |
| 259 | Japanese | 1177 | OK |
| 260 | Japanese | 1173 | OK |
| 261 | Japanese | 1173 | OK |
| 262 | Japanese | 1172 | OK |
| 263 | Japanese | 1169 | OK |
| 264 | Japanese | 1165 | OK |
| 265 | Japanese | 1156 | OK |
| 266 | Japanese | 1151 | OK |

**S1 Dataset.**

| Sr | Origin | Follow-up days | Outcome |
|---|---|---|---|
| 267 | Japanese | 1150 | OK |
| 268 | Japanese | 1148 | OK |
| 269 | Japanese | 1141 | OK |
| 270 | Japanese | 1138 | OK |
| 271 | Japanese | 1127 | OK |
| 272 | Japanese | 1123 | OK |
| 273 | Japanese | 1123 | OK |
| 274 | Japanese | 1121 | OK |
| 275 | Japanese | 1114 | OK |
| 276 | Japanese | 1114 | OK |
| 277 | Japanese | 1106 | OK |
| 278 | Japanese | 1102 | OK |
| 279 | Japanese | 1102 | OK |
| 280 | Japanese | 1466 | OK |
| 281 | Japanese | 1100 | OK |
| 282 | Japanese | 1092 | OK |
| 283 | Japanese | 1087 | OK |
| 284 | Japanese | 1081 | OK |
| 285 | Japanese | 1079 | OK |
| 286 | Japanese | 1072 | OK |
| 287 | Japanese | 1065 | OK |
| 288 | Japanese | 1052 | OK |
| 289 | Japanese | 1051 | OK |
| 290 | Japanese | 1050 | OK |
| 291 | Japanese | 1045 | OK |
| 292 | Japanese | 1043 | OK |
| 293 | Japanese | 1039 | OK |
| 294 | Japanese | 1037 | OK |
| 295 | Japanese | 1025 | OK |
| 296 | Japanese | 1024 | OK |
| 297 | Japanese | 1015 | OK |
| 298 | Japanese | 1005 | OK |
| 299 | Japanese | 1004 | OK |
| 300 | Japanese | 996 | OK |
| 301 | Japanese | 995 | OK |
| 302 | Japanese | 991 | OK |
| 303 | Japanese | 990 | OK |
| 304 | Japanese | 989 | OK |

**S1 Dataset.**

| Sr | Origin | Follow-up days | Outcome |
|---|---|---|---|
| 305 | Japanese | 983 | OK |
| 306 | Japanese | 980 | OK |
| 307 | Japanese | 976 | OK |
| 308 | Japanese | 961 | OK |
| 309 | Japanese | 961 | OK |
| 310 | Japanese | 959 | OK |
| 311 | Japanese | 956 | OK |
| 312 | Japanese | 953 | OK |
| 313 | Japanese | 953 | OK |
| 314 | Japanese | 947 | OK |
| 315 | Japanese | 934 | OK |
| 316 | Japanese | 931 | OK |
| 317 | Japanese | 928 | OK |
| 318 | Japanese | 926 | OK |
| 319 | Japanese | 925 | OK |
| 320 | Japanese | 920 | OK |
| 321 | Japanese | 920 | OK |
| 322 | Japanese | 919 | OK |
| 323 | Japanese | 919 | OK |
| 324 | Japanese | 919 | OK |
| 325 | Japanese | 918 | OK |
| 326 | Japanese | 917 | OK |
| 327 | Japanese | 913 | OK |
| 328 | Japanese | 912 | OK |
| 329 | Japanese | 912 | OK |
| 330 | Japanese | 911 | OK |
| 331 | Japanese | 911 | OK |
| 332 | Japanese | 906 | OK |
| 333 | Japanese | 903 | OK |
| 334 | Japanese | 892 | OK |
| 335 | Japanese | 889 | OK |
| 336 | Japanese | 885 | OK |
| 337 | Japanese | 883 | OK |
| 338 | Japanese | 865 | OK |
| 339 | Japanese | 864 | OK |
| 340 | Japanese | 862 | OK |
| 341 | Japanese | 862 | OK |
| 342 | Japanese | 854 | OK |

**S1 Dataset.**

| Sr | Origin | Follow-up days | Outcome |
|---|---|---|---|
| 343 | Japanese | 841 | OK |
| 344 | Japanese | 841 | OK |
| 345 | Japanese | 841 | OK |
| 346 | Japanese | 836 | OK |
| 347 | Japanese | 826 | OK |
| 348 | Japanese | 823 | OK |
| 349 | Japanese | 821 | OK |
| 350 | Japanese | 820 | OK |
| 351 | Japanese | 819 | OK |
| 352 | Japanese | 814 | OK |
| 353 | Japanese | 813 | OK |
| 354 | Japanese | 812 | OK |
| 355 | Japanese | 812 | OK |
| 356 | Japanese | 802 | OK |
| 357 | Japanese | 801 | OK |
| 358 | Japanese | 793 | OK |
| 359 | Japanese | 778 | OK |
| 360 | Japanese | 770 | OK |
| 361 | Japanese | 760 | OK |
| 362 | Japanese | 758 | OK |
| 363 | Japanese | 752 | OK |
| 364 | Japanese | 751 | OK |
| 365 | Japanese | 738 | OK |
| 366 | Japanese | 738 | OK |
| 367 | Japanese | 735 | OK |
| 368 | Japanese | 725 | OK |
| 369 | Japanese | 723 | OK |
| 370 | Japanese | 723 | OK |
| 371 | Japanese | 714 | OK |
| 372 | Japanese | 710 | OK |
| 373 | Japanese | 701 | OK |
| 374 | Japanese | 696 | OK |
| 375 | Japanese | 694 | OK |
| 376 | Japanese | 675 | OK |
| 377 | Japanese | 669 | OK |
| 378 | Japanese | 668 | OK |
| 379 | Japanese | 668 | OK |
| 380 | Japanese | 667 | OK |

**S1 Dataset.**

| Sr | Origin | Follow-up days | Outcome |
|---|---|---|---|
| 381 | Japanese | 660 | OK |
| 382 | Japanese | 659 | OK |
| 383 | Japanese | 655 | OK |
| 384 | Japanese | 654 | OK |
| 385 | Japanese | 653 | OK |
| 386 | Japanese | 648 | OK |
| 387 | Japanese | 646 | OK |
| 388 | Japanese | 645 | OK |
| 389 | Japanese | 645 | OK |
| 390 | Japanese | 641 | OK |
| 391 | Japanese | 640 | OK |
| 392 | Japanese | 623 | OK |
| 393 | Japanese | 623 | OK |
| 394 | Japanese | 609 | OK |
| 395 | Japanese | 604 | OK |
| 396 | Japanese | 603 | OK |
| 397 | Japanese | 598 | OK |
| 398 | Japanese | 598 | OK |
| 399 | Japanese | 585 | OK |
| 400 | Japanese | 585 | OK |
| 401 | Japanese | 584 | OK |
| 402 | Japanese | 584 | OK |
| 403 | Japanese | 582 | OK |
| 404 | Japanese | 571 | OK |
| 405 | Japanese | 569 | OK |
| 406 | Japanese | 563 | OK |
| 407 | Japanese | 563 | OK |
| 408 | Japanese | 555 | OK |
| 409 | Japanese | 555 | OK |
| 410 | Japanese | 554 | OK |
| 411 | Japanese | 550 | OK |
| 412 | Japanese | 543 | OK |
| 413 | Japanese | 541 | OK |
| 414 | Japanese | 541 | OK |
| 415 | Japanese | 540 | OK |
| 416 | Japanese | 527 | OK |
| 417 | Japanese | 525 | OK |
| 418 | Japanese | 522 | OK |

**S1 Dataset.**

| Sr | Origin | Follow-up days | Outcome |
|---|---|---|---|
| 419 | Japanese | 522 | OK |
| 420 | Japanese | 521 | OK |
| 421 | Japanese | 514 | OK |
| 422 | Japanese | 514 | OK |
| 423 | Japanese | 508 | OK |
| 424 | Japanese | 504 | OK |
| 425 | Japanese | 501 | OK |
| 426 | Japanese | 499 | OK |
| 427 | Japanese | 498 | OK |
| 428 | Japanese | 487 | OK |
| 429 | Japanese | 476 | OK |
| 430 | Japanese | 471 | OK |
| 431 | Japanese | 469 | OK |
| 432 | Japanese | 464 | OK |
| 433 | Japanese | 463 | OK |
| 434 | Japanese | 462 | OK |
| 435 | Japanese | 459 | OK |
| 436 | Japanese | 456 | OK |
| 437 | Japanese | 448 | OK |
| 438 | Japanese | 438 | OK |
| 439 | Japanese | 428 | OK |
| 440 | Japanese | 427 | OK |
| 441 | Japanese | 423 | OK |
| 442 | Japanese | 409 | OK |
| 443 | Japanese | 406 | OK |
| 444 | Japanese | 396 | OK |
| 445 | Japanese | 393 | OK |
| 446 | Japanese | 392 | OK |
| 447 | Japanese | 389 | OK |
| 448 | Japanese | 388 | OK |
| 449 | Japanese | 378 | OK |
| 450 | Japanese | 378 | OK |
| 451 | Japanese | 375 | OK |
| 452 | Japanese | 193 | Police |
| 453 | Japanese | 124 | Police |
| 454 | Japanese | 658 | Police |
| 455 | Japanese | 998 | Referred |
| 456 | Japanese | 938 | Referred |

**S1 Dataset.**

| Sr | Origin | Follow-up days | Outcome |
|---|---|---|---|
| 457 | Japanese | 1128 | Referred |
| 458 | Japanese | 594 | Referred |
| 459 | Japanese | 778 | Referred |
| 460 | Japanese | 749 | Referred |
| 461 | Japanese | 436 | Referred |
| 462 | Japanese | 982 | Referred |
| 463 | Japanese | 694 | Referred |
| 464 | Japanese | 1500 | Referred |
| 465 | Japanese | 20 | Referred |
| 466 | Japanese | 316 | Referred |
| 467 | Japanese | 869 | Referred |
| 468 | Japanese | 511 | Referred |
| 469 | Japanese | 184 | Referred |
| 470 | Japanese | 1423 | Referred |
| 471 | Japanese | 1114 | Referred |
| 472 | Japanese | 334 | Referred |
| 473 | Japanese | 77 | Referred |
| 474 | Japanese | 379 | Referred |
| 475 | Japanese | 1317 | Referred |
| 476 | Japanese | 172 | Referred |
| 477 | Japanese | 290 | Referred |
| 478 | Japanese | 829 | Referred |
| 479 | Japanese | 18 | Referred |
| 480 | Japanese | 8 | Referred |
| 481 | Japanese | 322 | Referred |
| 482 | Japanese | 40 | Referred |
| 483 | Japanese | 8 | Referred |
| 484 | Japanese | 857 | Referred |
| 485 | Japanese | 738 | Referred |
| 486 | Japanese | 61 | Referred |
| 487 | Japanese | 42 | Referred |
| 488 | Japanese | 358 | Referred |
| 489 | Japanese | 354 | Referred |
| 490 | Japanese | 1066 | Referred |
| 491 | Japanese | 596 | Referred |
| 492 | Japanese | 667 | Referred |
| 493 | Japanese | 148 | Referred |
| 494 | Japanese | 464 | Referred |

**S1 Dataset.**

| Sr | Origin | Follow-up days | Outcome |
|---|---|---|---|
| 495 | Japanese | 463 | Referred |
| 496 | Japanese | 221 | Referred |
| 497 | Japanese | 264 | Referred |
| 498 | Japanese | 169 | Referred |
| 499 | Japanese | 36 | Referred |
| 500 | Japanese | 190 | Referred |
| 501 | Japanese | 303 | Referred |
| 502 | Japanese | 621 | Referred |
| 503 | Japanese | 995 | Referred |
| 504 | Japanese | 869 | Referred |
| 505 | Japanese | 365 | Referred |
| 506 | Japanese | 352 | Referred |
| 507 | Japanese | 357 | Referred |
| 508 | Japanese | 503 | Referred |
| 509 | Japanese | 34 | Referred |
| 510 | Japanese | 154 | Referred |
| 511 | Japanese | 56 | Referred |
| 512 | Japanese | 970 | Referred |
| 513 | Japanese | 148 | Referred |
| 514 | Japanese | 404 | Referred |
| 515 | Japanese | 141 | Referred |
| 516 | Japanese | 29 | Referred |
| 517 | Japanese | 208 | Referred |
| 518 | Japanese | 1018 | Referred |
| 519 | Japanese | 101 | Referred |
| 520 | Japanese | 130 | Referred |
| 521 | Japanese | 792 | Referred |
| 522 | Japanese | 540 | Referred |
| 523 | Japanese | 29 | Referred |
| 524 | Japanese | 419 | Referred |
| 525 | Japanese | 1 | Referred |
| 526 | Japanese | 79 | Referred |
| 527 | Japanese | 184 | Referred |
| 528 | Japanese | 470 | Referred |
| 529 | Japanese | 790 | Referred |
| 530 | Japanese | 428 | Referred |
| 531 | Japanese | 324 | Referred |
| 532 | Japanese | 161 | Referred |

**S1 Dataset.**

| Sr | Origin | Follow-up days | Outcome |
|---|---|---|---|
| 533 | Japanese | 36 | Referred |
| 534 | Japanese | 43 | Referred |
| 535 | Japanese | 8 | Referred |
| 536 | Japanese | 95 | Referred |
| 537 | Japanese | 36 | Referred |
| 538 | Japanese | 356 | Referred |
| 539 | Japanese | 225 | Referred |
| 540 | Japanese | 6 | Referred |
| 541 | Japanese | 56 | Referred |
| 542 | Japanese | 22 | Referred |
| 543 | Japanese | 319 | Referred |
| 544 | Japanese | 196 | Referred |
| 545 | Japanese | 164 | Referred |
| 546 | Japanese | 86 | Referred |
| 547 | Japanese | 150 | Referred |
| 548 | Japanese | 357 | Referred |
| 549 | Japanese | 41 | Referred |
| 550 | Japanese | 169 | Referred |
| 551 | Japanese | 324 | Referred |
